# Exogenous Ochronosis: Characterizing a Rare Disorder in Skin of Color

**DOI:** 10.3390/jcm12134341

**Published:** 2023-06-28

**Authors:** Michelle Lazar, Henriette De La Garza, Neelam A. Vashi

**Affiliations:** Department of Dermatology, Boston University School of Medicine, 609 Albany St, J502, Boston, MA 02118, USA; lazar4@bu.edu (M.L.); henriettedlg@gmail.com (H.D.L.G.)

**Keywords:** exogenous ochronosis, skin of color, lasers, microneedling, ethnic skin, hydroquinone

## Abstract

Exogenous ochronosis is a rare dyschromia that primarily impacts those with skin of color. It is characterized by blue–black pigmentation and is associated with the long-term application of skin-lightening creams containing hydroquinone. Commonly confused with other dyschromias, the use of skin lightening topicals can cause paradoxical skin darkening in patients with known exogenous ochronosis. This is highly distressing to patients, often worsening the underlying dyschromia and making treatment more difficult. A 10-year retrospective analysis was conducted that revealed 25 patients with exogenous ochronosis. The average patient used a skin lightening cream for 9.2 years, with exogenous ochronosis most commonly arising on the cheeks (68%), forehead (24%), and temples (20%). Furthermore, this study identified that patients with exogenous ochronosis may respond well to treatment with Q-switched Alexandrite laser and microneedling. The incidence of exogenous ochronosis is likely to increase as demographics shift and access to a wide range of over-the-counter topicals becomes more available, both in the United States and worldwide. Therefore, it is imperative to better characterize exogenous ochronosis to identify best treatment practices for all patients.

## 1. Introduction

Exogenous ochronosis (EO) is a cutaneous disorder characterized by blue–black pigmentation that is thought to be a complication of the long-term application of skin-lightening creams containing, in particular, hydroquinone (HQ). This paradoxical hyperpigmentation that is thought to occur with the use of skin lightening agents is very distressing to many patients and can easily be mistaken for other forms of dyschromia, such as melasma, post-inflammatory hyperpigmentation, and Riehl’s melanosis, amongst others. EO predominantly occurs in darker skinned individuals and is thought to occur due to the deposition of homogentisic acid in the skin, although the exact mechanism is still unknown [1]. It has been surmised that the risk of EO increases if the concentration of HQ is greater than 2%, especially when in an alcoholic solution [1]. There are a variety of reasons that patients utilize HQ compounds or other skin lightening topicals. However, what makes EO such a difficult disease to manage is that the hyperpigmentation is exactly contrary to the original use of the products that lead to the disease. As a result, many patients utilize higher concentrations of HQ or apply HQ compounds more regularly, only worsening their EO. Therefore, given the difficulty of diagnosis, many patients continue to utilize hydroquinone-containing products not knowing that EO has already occurred and is causal to their worsening pigmentary alteration [2]. This disorder can be greatly distressing to patients as typical treatments for other hyperpigmentation disorders have difficulty alleviating the condition.

The current incidence of EO is not well known due to the esoteric nature of the disorder [1]. One of the largest case series studied to date was in a South African population and is where much of the early data for the disease originated [1]. Data from the United States Department of Health and Human Services reported 512 suspected cases of EO in the United States [2]. However, it is believed that EO is underreported in the US, for a myriad of reasons [2]. These include the rare nature of the disease, but also the difficulty distinguishing EO from other common dyschromias. Furthermore, many patients may have EO but not seek care for the condition, leading to further underreporting of the disease.

Once identified, EO can be classified into three stages to track progression, as outlined by Dogliotti and Leibowitz [3]. Stage I is characterized by erythema and mild hyperpigmentation. Following this, stage II consists of hyperpigmentation, black colloid milia (so called caviar-like lesions), and atrophy. Finally, stage III includes papulo-nodular lesions. This system allows for the categorization of initial disease presentation, as well as the tracking of natural disease progression and intervention effects. There are other methods of classification used less commonly, such as those described by Phillips in 1986, with a scale of mild, moderate, and severe, and Hardwick in 1989. with a grade of I through V [1].

EO is still a poorly characterized disorder and often misdiagnosed at first presentation, mostly due to the difficulty many have in distinguishing EO from other hyperpigmentation disorders. Currently, the gold standard for EO diagnosis is biopsy-proven analysis. Histology will show evidence of ochronotic fibers under microscopy [2]. Biopsy is the gold standard as EO can co-occur with other dyschromic conditions and biopsy analysis allows for the resolute diagnosis of EO. However, dermoscopy is an underused tool that can aid in diagnosis, possibly avoiding the need for biopsy. In dermoscopy, the obliteration of follicular openings has been found to correlate with biopsy-proven histological findings of EO [2]. As a result, if patients have findings consistent with EO, both in oral history and dermoscopy presentation, providers may be able to avoid the biopsy of a highly sensitive facial area. However, as previously mentioned, melasma and EO can co-exist; thus, caution must be taken to ensure a thorough investigation of all dyschromic areas is made, as one diagnosis does not preclude the other. 

EO is a disorder that primarily occurs in skin of color. The Fitzpatrick skin type system (FST) is commonly used in dermatology to classify skin tone and color in a standardized manner. Type I skin always burns, never tans and is the least pigmented of all skin types. Type VI skin never burns and is the deepest level of pigmentation. Skin exists on a spectrum of pigmentation, and the FST system seeks to allow for standardization of base pigmentation assessment. As a result of the nature of EO, the majority of patients are FST III through VI. However, EO should not be excluded from the differential diagnosis in a patient with an FST of I-II if the clinical picture is concerning for EO. Furthermore, the appearance of EO may vary depending on the patient’s base skin color. Therefore, identifying a patient’s FST by utilizing areas of unaffected skin can help prior to the exam. 

Disorders such as EO need to be characterized and recognized given the difficulty of treatment and the permanent nature of skin changes. Disorders that affect primarily darker skinned individuals will only become more important as the demographic of the United States (US) shifts. It is predicted that by 2045, non-Hispanic Whites will no longer constitute the majority of the population [4]. Disorders of pigmentation, which occur more frequently in the skin of color population, need to be fully understood and recognized to ensure proper diagnosis and therapeutic intervention. Specifically, it is vital to better understand rare disorders, such as EO, to provide effective treatment modalities for patients of all skin types. Through our study, we sought to better characterize EO and evaluate the outcomes of different treatments in our cohort of patients.

## 2. Materials and Methods

A retrospective chart review was conducted on patients who received care at Boston Medical Center (BMC) and who had medical records that contained mention of EO; these were collated by the BMC Clinical Data Warehouse. This search included all patients who had the terms “ochronosis”, “exogenous ochronosis”, E270.2, or E70.29 billing codes in their records. Exclusion criteria included anyone under the age of 18, those who were incarcerated, and other vulnerable populations. This search resulted in 34 total patients. Each medical record was then reviewed by the study team. Final analysis resulted in 25 patients who had confirmed EO after chart review, either by final diagnosis or histopathological confirmation.

## 3. Results

This study was approved by the Boston University and Boston Medical Center IRB. A 10-year retrospective analysis was conducted that revealed 25 patients with EO, with over 50% having biopsy confirmation.

Our sample consisted of 22 females and 3 males with an average age of 55; the youngest age at diagnosis was 33 and the oldest age at diagnosis was 69 (Table 1). Approximately, 28% were skin type IV and 64% were skin type V/VI. Eighteen patients regarded themselves as Black/African American, one as Asian, and one as White. Of the patients whose country of origin was known, patients were from: Cape Verde (4), Haiti (4), the Democratic Republic of Congo (2), Congo (2), Benin (1), Nigeria (1), Trinidad (1), and Uganda (1).

Sunscreen usage was variable; from available data, nine patients (36%) reported the use of sun protection. The majority of patients did not know or could not quantify their use of sunscreen or sun protection. Additionally, most patients (84%) used over-the-counter topicals and were told of the association with their EO. Of all patients, 16 (64%) were aware that the cream incorporated a bleaching agent and used the product regardless. Six patients (24%) had been using a 2% HQ formulation; the remainder used products whose HQ percentage and general formulation was not known. The average length of use of the offensive topical was 9.2 years. The shortest duration of use of a product was 1.5 years and was of an unknown formulation. The longest duration of use of a product was 20 years and was a 2% HQ-containing compound. The most common locations for EO were: cheeks (68%), forehead (24%), and temples (20%). Sixteen patients (64%) had EO noted in multiple areas. The most common description of EO as noted in the medical record were: “gray” (44%), “brown” (32%), “black” (24%), and “blue” (16%). Of the patients in this analysis, eight (32%) were misdiagnosed at their first arrival, with six being misdiagnosed originally as melasma by both dermatologist and non-dermatologist providers.

**Figure 1:**
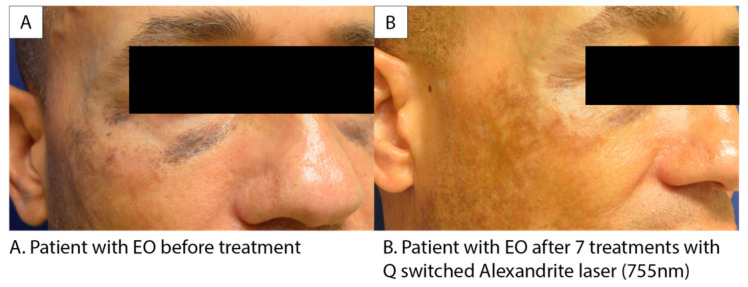
Patient images before and after treatment with the Q-switched Alexandrite laser.

In our cohort, treatment modalities included: tretinoin (6), microneedling (5), laser (5), Ambifade (1), Triluma (4), compound treatment with steroid, HQ, and tretinoin (1), hydrocortisone (1), ketoconazole (1), ammonium lactate (1), tranexamic acid (1), Melquin (1), and Esoterica (1). Some treatments were offered secondary to initial misdiagnosis, and many patients received multiple treatment modalities. Of these treatments, microneedling and Q-switched Alexandrite laser were the most successful treatments with three patients in each responding favorably to the procedures. Those who had favorable responses to microneedling had an average of 2.33 treatments. Patients who had favorable responses to the laser treatments had an average of 4.66 total treatments. Figure 1 showcases a favorable response to Alexandrite treatment in a patient with EO, as indicated by a reduction in the level of dyschromia. Topical options were not found to be helpful, including but not limited to: Triluma, Tretinoin, and ammonium lactate. While these treatment options often help treat dyspigmentation due to melasma, they are not effective against EO.

## 4. Discussion

Hydroquinone remains the gold standard for hyperpigmentation therapy in the US, which has typically been available at 2% over the counter (OTC) and various prescription percentages [5]. This has allowed patients who are not able to afford prescription HQ compounds to have access to compounds that can help alleviate their symptoms, depending on the origin of their hyperpigmentation. Conversely, the use of these products can result in damage to the skin, resulting in the development of EO; largely, this occurs due to lack of appropriate knowledge on adverse effects associated with prolonged use. However, during the COVID-19 pandemic, the CARES Act was passed, which resulted in all OTC HQ being taken off the market in the United States [6]. This change in legislation aligns with international regulations similar to those seen in the European Union, which has prohibited the use of HQ in cosmetic compounds since 2009 [7]. Similarly, HQ is banned in OTC cosmetic products in Ghana, Australia, and Japan [8,9]. Nevertheless, many other international markets do not regulate HQ—resulting in international variation in supply, purity, and concentration of HQ compounds available.

As a result of the CARES Act, patients in the United States will now have reduced access to topical lightening creams, especially those containing HQ. While this could lead to a theoretical reduction in the incidence of EO, there is a concern that if patients do not have access to OTC HQ compounds in the United States, they will obtain these products from other countries where HQ is less strictly regulated [10]. Decreased oversight in overseas markets can lead to higher levels of impurities or toxic substances. This was highlighted in a study conducted on West African and Canadian lightening creams, which found that 38% (37/98) of creams analyzed surpassed the impurity threshold set by the European Union legislation [11]. Therefore, while a reduction in access to OTC HQ in the United States may be well-intentioned, there is significant risk if patients continue to use HQ-containing products from other countries that are less regulated and therefore less safe.

Interestingly, even though most of the patients in this study were diagnosed prior to the passage of the CARES Act, none of the patients in this study had acquired their HQ cream from a medical doctor in the US. As a result, much of the EO that was identified in this study occurred due to use of OTCs or prescription products acquired in other countries. Increased accessibility paired with unsupervised usage of HQ heightens the risk of EO development, especially in patients who do not engage in sun protective behaviors.

For the majority of patients, the development of EO is distressing as the hyperpigmentation is the exact inverse of the desired effect that initially led to the utilization of HQ-containing OTCs creams. Therefore, many patients seek other treatments to reduce the dyschromia. It has previously been found that six patients, with varying severity of EO, responded well to Q-switched Nd:YAG laser treatments [12]. We demonstrated efficacy with Q-switched Alexandrite laser treatments for EO as highlighted in Figure 1. There is a noticeable reduction in the level of dyschromia following treatment. For patients who struggle with EO, laser-based treatment options should be explored to help lessen the hyperpigmentation and thus the severity of the EO, especially in prominent facial regions. Q-switched Alexandrite laser treatments have been proven to be effective and safe in carefully selected skin of color patients, with no increase in rates of adverse events when utilized properly [13]. Therefore, this modality may be utilized more in EO due to the patient population impacted.

Additionally, the use of microneedling as a treatment modality for EO has not been studied previously, but our study identifies this as a potential treatment option in the future. This is the first report demonstrating its efficacy in EO. Microneedling ought to be utilized further as it is a treatment modality with fewer adverse events in those with darker skin types [14]. The utilization of microneedling could be as an adjunct or instead of Q-switched Alexandrite treatment in certain patients. As microneedling has been shown to be effective in skin of color with fewer adverse events, utilizing it in the treatment of EO has a two-fold benefit; it improves the appearance of EO, and microneedling has a lesser side effect profile when compared to many other treatments in skin of color. Microneedling could therefore help offset the distress that comes with an EO diagnosis as well as preventing further distress from the treatment of adverse events.

The literature has noted that some patients with EO have responded to topical retinoic acid, glycolic acid, or topical corticosteroids [1]. We did not see these same effects in our study population; however, for certain subsets of EO, it may be a useful treatment modality prior to more invasive procedures. Furthermore, chemical peeling and derma-abrasion may also be useful in a subset of patients [1]. These modalities were not utilized in our study population and thus we cannot comment on their efficacy in our cohort; however, these treatment strategies ought to be further studied to identify if they can be used as alternative treatments for EO. 

Limitations of this study include the single center retrospective design. As a result, the patients analyzed in this data set may not be indicative of the greater patient population pool of all those with EO. Furthermore, as this is a retrospective analysis, there is an inability to directly compare treatment modalities as if it was a randomized control trial. Finally, the sample size, while one of the larger EO studies conducted, is small and therefore may not have enough power to effectively mirror worldwide patterns.

Further research needs to be conducted to identify the best protocols to follow when treating EO; however, laser treatment and microneedling may provide benefit to patients who have not had satisfactory responses to other treatment modalities. As the population demographics of the United States begin to shift, we must ensure we have appropriate treatments for patients of all demographic backgrounds and skin colors.

## Figures and Tables

**Table 1:** Demographic information.

Race	Number (*n* = 25) (%)	Sex	Number (*n* = 25) (%)	Treatment Modalities	Number (*n* = 25)
Black/African American	18 (72.00%)	Male	3 (12.00%)	Tretinoin	6 (24.00%)
White	1 (4.00%)	Female	22 (88.00%)	Microneedling	5 (20.00%)
Asian	1 (4.00%)	**Age Range**		Laser	5 (20.00%)
Declined/NA	5 (20.00%)	Minimum	33	Triluma	4 (16.00%)
**Ethnicity**		Max	69	Ambifade	1 (4.00%)
Hispanic	1 (4.00%)	Mode	53	Ammonium Lactate	1 (4.00%)
Non-Hispanic	24 (96.00%)	**Location(s)**		Compound cream	1 (4.00%)
**Country of Origin**		Multiple	16 (64.00%)	Esoterica	1 (4.00%)
Cape Verde	4 (16.00%)	Cheeks	17 (68.00%)	Hydrocortisone	1 (4.00%)
Haiti	4 (16.00%)	Forehead	6 (24.00%)	Ketoconazole	1 (4.00%)
DRC	2 (8.00%)	Temples	5 (20.00%)	Melquin	1 (4.00%)
Congo	2 (8.00%)	Periocular	4 (16.00%)	Tranexamic acid	1 (4.00%)
Benin	1 (4.00%)	Neck	3 (12.00%)		
Nigeria	1 (4.00%)	Periorbital	3 (12.00%)		
Trinidad	1 (4.00%)	Infraorbital	3 (12.00%)		
Uganda	1 (4.00%)	**Known Use of Damaging Product**			
**FST**		Yes	21 (84.00%)		
I	0	No	1 (4.00%)		
II	0	Unknown	3 (12.00%)		
III	0	**Known Use of Bleaching Agent**			
IV	7 (28.00%)	Yes	16 (64.00%)		
V	10 (40.00%)	No	1 (4.00%)		
VI	6 (24.00%)	Unknown	8 (32.00%)		
N/A	2 (8.00%)				

## Data Availability

Data are not available due to the small cohort of patients and rare nature of the disease for patient privacy.
